# Diamond-like carbon coating under oleic acid lubrication: Evidence for graphene oxide formation in superlow friction

**DOI:** 10.1038/srep46394

**Published:** 2017-04-12

**Authors:** Maria Isabel De Barros Bouchet, Jean Michel Martin, José Avila, Makoto Kano, Kentaro Yoshida, Takeshi Tsuruda, Shandan Bai, Yuji Higuchi, Nobuki Ozawa, Momoji Kubo, Maria C. Asensio

**Affiliations:** 1 University of Lyon, Ecole Centrale de Lyon, LTDS, 69380, Ecully, France; 2 ANTARES Beamline, Synchrotron SOLEIL Université Paris-Saclay, L’Orme des Merisiers, Saint Aubin-BP 48, 91192 Gif sur Yvette Cedex, France; 3 Kanagawa Industrial Technology Center 705-1 Shimo-Imaizumi, Ebina, Kanagawa, Japan; 4 Institute for Materials Research, Tohoku University, 2-1-1 Katahira, Aoba-ku, Sendai 980-8577, Japan; 5 New Industry Creation Hatchery Center, Tohoku University, 6-6-10 Aoba, Aramaki, Aoba-ku, Sendai 980-8579, Japan

## Abstract

The achievement of the superlubricity regime, with a friction coefficient below 0.01, is the Holy Grail of many tribological applications, with the potential to have a remarkable impact on economic and environmental issues. Based on a combined high-resolution photoemission and soft X-ray absorption study, we report that superlubricity can be realized for engineering applications in bearing steel coated with ultra-smooth tetrahedral amorphous carbon (ta-C) under oleic acid lubrication. The results show that tribochemical reactions promoted by the oil lubrication generate strong structural changes in the carbon hybridization of the ta-C hydrogen-free carbon, with initially high sp^3^ content. Interestingly, the macroscopic superlow friction regime of moving mechanical assemblies coated with ta-C can be attributed to a few partially oxidized graphene-like sheets, with a thickness of not more than 1 nm, formed at the surface inside the wear scar. The sp^2^ planar carbon and oxygen-derived species are the hallmark of these mesoscopic surface structures created on top of colliding asperities as a result of the tribochemical reactions induced by the oleic acid lubrication. Atomistic simulations elucidate the tribo-formation of such graphene-like structures, providing the link between the overall atomistic mechanism and the macroscopic experimental observations of green superlubricity in the investigated ta-C/oleic acid tribological systems.

The reduction of the energy loss by mechanical friction especially for automotive engines has been an important requirement in recent years for improving fuel economy and providing a solution to environmental issues. Friction and wear remain the primary routes of mechanical energy dissipation in general mechanical assemblies. It is estimated that approximately a third of the fuel in automobiles is only used to overcome friction^1^. Diamond-like carbon (DLC) is a remarkable metastable form of amorphous carbon with a significant amount of sp^3^ bonds; DLC is characterized by a wide band gap, semiconductive properties, high mechanical hardness, chemical inertness and optical transparency^2^,^3^,^4^,^5^,^6^. DLC films have a widespread application as protective coatings in areas as diverse as optical windows, magnetic storage disks, car engine parts, biomedical coatings and micro-electromechanical systems (MEMS)^7^,^8^,^9^,^10^. Moreover, DLC coatings exist as a wide variety of amorphous, flexible, and purely and partially sp^3^ bonded diamond-like hybridized carbon atoms. The hardest and strongest type of DLC is the ta-C, which is considered the “pure” form of DLC because it consists mainly of sp^3^-bonded carbon atoms in an amorphous structure.

However, to create superlubricity regimes, both DLC and ta-C thin films need to be optimized by doping the bare materials with fillers, such as hydrogen, a large variety of metals and graphitic carbon with sp^2^ bonds, to obtain coatings with an optimal amount of sp^3^/sp^2^ bondings^7^,^8^,^9^,^10^. Although DLC coatings have been increasingly utilized in widespread applications, diamond-like carbon and related coatings should be used in many highly stressed mechanical components, including bearing and gears. Several friction tests have shown an increase in the load carrying capacity and a prolonged lifetime when the DLC coatings are partially hydrogenated or doped with metals^10^,^11^,^12^,^13^. However, the simplest method to control wear is by utilizing better lubricants. Although the power densities in gear transmissions always increase with a corresponding increase in the gear oil stresses, and the mechanical load and the work temperature increase as the lubricating film thickness reduces, the lubricant formulations can be well controlled, and they consequently induce beneficial tribological reactions that can stabilize superlow friction regimes. In particular, the superlow friction property, with a friction coefficient below 0.01, is the most outstanding advantage of DLC coatings used in vacuum or inert gas^14^,^15^. However, there are no current successful industrial accomplishments in this respect except in space applications. Remarkably, DLC-coated valve lifters and piston rings have been recently applied to reduce engine friction for mass-produced gasoline engines in Japan^16^,^17^. The application of DLC coatings because of their low friction property is the first case that involves using hydrogen-free DLC, with theoretically full sp^3^ hybridized carbon atoms (ta-C) lubricated by an ester-containing gasoline engine oil^18^. Additionally, the ultra-low friction properties obtained by using ta-C were also observed under lubrication by glycerol and oleic acid, although the hydrogenated DLC (a-C:H) did not show such a low friction property^19^,^20^,^21^,^22^,^23^. DLC coatings combined with selected green lubricants can be used in many other industrial fields including biological and food applications.

In recent reports, tribochemical reactions that lead to the production of functional groups such as –OH and –COOH on the sliding surfaces of ta-C and a-C:H have been proposed. However, the nature and manner through which the tribofilm should be built is under debate^7^,^8^,^9^,^10^. The aim of this work is to report on a compelling study of the nature and chemistry of the tribofilm formed by moving mechanical assemblies under oil lubrication. We obtain deeper insight at the nanometre scale and infer the underlying superlow friction mechanism. Our findings show the existence of a superlubricity regime in self-mated DLCs under the application of oleic acid as a lubricant. More interestingly, through a complementary study of high–resolution synchrotron radiation photoemission (HRPES) together with soft X-ray absorption near edge structure (XANES), we demonstrate that superlubricity can be realized for engineering applications when ta-C coatings are lubricated by fatty oleic acid owing to the presence of a partially oxidized ultrathin graphene-like film. This protective film is produced by the tribochemical reactions that take place at the surface under friction. Theoretical simulations were also used to investigate the atomistic origin of the protective film, shedding light on the chemical reactions that occur at the tribofilm. To date, this is the first reported case of *in situ* formation of graphene oxide species directly induced by friction.

## Results

### Friction results

**Figure 1:**
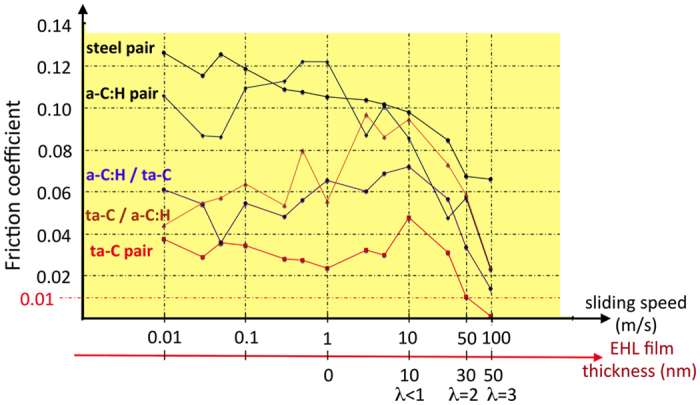
Friction results of decreasing sliding speed tests with different friction DLC pairs lubricated by pure oleic acid at ambient temperature and RH 45%. For ta-C pairs, EHL conditions (λ > 3) are reached, as shown with the red circle, and ML conditions (1 < λ < 3) are shown with the dashed red circle. Only the ta-C friction pair shows superlubricity. The error bars (not represented for clarification) are about ±0.005 for friction values between 0.04 and 0.1 and ±0.003 for CoF below 0.01, respectively.

Figure 1 compares the friction results for the four possible DLC configurations under oleic acid lubrication at ambient temperature (RH of 45%); the test results were obtained by decreasing the sliding speed step by step from 100 mm/s to 0.01 mm/s. The results obtained for steel/steel friction pair are also presented on the figure for comparison. Each friction experiment was realised three times and reproducibility was quite good. The values in the curves are the average value of these three measurements. These results are shaped like a Stribeck curve, and they show the different regimes that are typically encountered in such a lubrication test, namely, elasto-hydrodynamic lubrication (EHL) above 100 mm/s, boundary lubrication (BL) under 10 mm/s and mixed lubrication (ML) between these two speeds. Typically, the superlubricity regime is defined by friction coefficient values below 0.01^24^. Under severer boundary lubrication conditions, superlubricity has never been attained so far, but CoF inferior to 0.04 is abnormally low for the boundary regime.

Clearly, amazing friction results were obtained for the ta-C-coated pair, and we will focus on this case in the following section. At all speeds, the friction coefficients under oleic acid lubrication for the ta-C pair were much lower than the friction coefficients for the a-C:H pair as well as for the mixed ta-C/a-C:H and a-C:H/ta-C combinations. The ta-C coating is obtained by filtered PVD deposition technique and it does not need any polishing after deposition. It is as smooth as the hydrogenated DLC coating, a-C:H (see test method in additional information section). The ta-C thickness is close to 300 nm and has been determined using transmission electron microscopy (TEM) on a sample transversal section nanomachined by FIB (focused ion beam). In comparison, the steel/steel pair displays the worst behaviour. The superlow friction coefficient of approximately 0.005 for ta-C above a 50 mm/s sliding speed belongs to the mixed lubrication regime. We have calculated the EHL film thickness using Dowson’s equation^25^ equations and we have also reported in Fig. 1 some lambda values of interest (ratio between film thickness and composite roughness of the two surfaces). Indeed, a calculation of the minimum film thickness in the contact zone at 50 mm/s speed gives a value of approximately 20 nm and a lambda ratio of 2 (the film thickness divided by the composite roughness of the two surfaces, *i.e*., 10 nm in our case).

**Figure 2:**
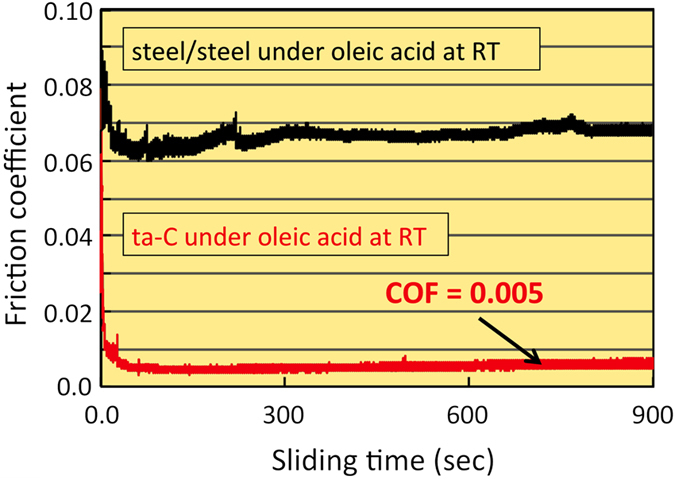
Friction results at constant sliding speed tests with ta-C pairs and oleic acid at ambient temperature featuring ML conditions with superlow friction at 66% RH. PES and XAS will be performed at the end of this test and compared inside and outside the cylinder wear scar.

An example of superlubricity under the mixed lubrication regime and constant sliding speed is shown in Fig. 2 for a ta-C friction pair at a constant sliding speed of 50 mm/s, a mean contact pressure of 100 MPa and a RH of 66%. The friction coefficient starts at 0.1 and drops drastically below 0.01 after a test lasting a few tens of seconds long. Afterwards, the regime remained at this very low CoF value for at least 900 s. The friction value is in good agreement with the results displayed in Fig. 1, from the decreasing speed test. As shown in Fig. 2, such a remarkable superlow friction coefficient is absolutely not observed for the traditional steel/steel pair under same lubrication conditions and with same surface roughness. So, this remarkable behaviour cannot be only imputed to a transition through mixte/EHL regimes and the surface chemistry is certainly involved.

In a previous work^26^, we emphasized the role of the OH-termination of amorphous carbon on the superlow friction values using ToF-SIMS surface analysis with deuterated oleic acid as the lubricant. However, we had no information on the crystal structure of the outmost surface in that study. So, to obtain high-resolution chemical and electronic information we need to use techniques with an extremely high surface sensitivity such as the synchrotron radiation-based methods used in this work. These techniques ensure the preferential detection of chemical species present at the topmost atomic surface layers. We preferred Photo-Emission Spectroscopy (PES) to other potential techniques like Raman spectroscopy because of the depth resolution that is more accurate in the case of superimposed carbon-rich layers with possible gradient of hybridization.

In this work, our target is to chemically characterize the very low shear strength of the tribofilm on the carbon top surface that leads to a friction coefficient below 0.01. The optical images of the sliding surfaces show that the ta-C coating on the disc and cylinder are not delaminated during the test. Only a slight change of colour allows for a definite detection of the contacting area owing to a slight reduction in the coating thickness from the clipping of the colliding asperities. Generally, the observation of the residual lubricant on the disc after removing the cylinder is interesting. In the case of ta-C, the residual lubricant wets the worn surface at the inside the wear scar, which implies that the worn area has become partially hydrophilic after the test^26^.

### Superlow friction mixed regime investigated by high-resolution photoemission spectroscopy and soft X-ray absorption analyses

Extreme surface sensitivity is needed to probe the electronic and chemical composition before and after the controlled friction tests. We have combined variable incident photon energy PES with soft XAS to record the “fingerprints” of carbon- and oxygen-derived species before and after the tribochemical reactions that occur at the topmost surface atomic layers. The coupling of the two techniques reveals the degree of carbon hybridization with a very high depth resolution (less than 1 nm), as well as the distribution of the existing species on the surface using the mesoscopic lateral spatial resolution; this allows us to perfectly distinguish spectroscopic chemical information from inside and outside the wear scars (see photoemission spectroscopies in additional information section).

**Figure 3:**
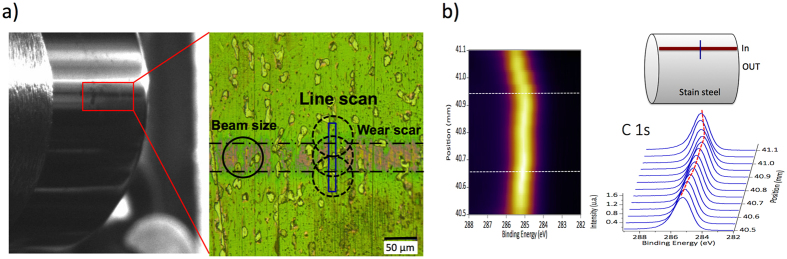
(**a**) optical micrographs of the worn ta-C coated cylinder after the test under thin film mixed ML conditions and details of the wear scar on the generatrix of the cylinder. The size of the X-ray beam for surface analysis is also shown. (**b**) Line-scan analysis on the C1s photopeak across the wear scar in the middle of the cylinder. Inside the wear scar, the shift of the C1s peak to a lower energy is clearly visible.

Figure 3a shows an optical image of the entire cylinder, used for the superlow friction test under the ML conditions reported in Fig. 2, together with a schematic indication of the wear scar located on the generatrix of the cylinder and the X-ray spot size utilized. The width of the wear scar is approximately 50 microns, almost corresponding to the calculated Hertzian contact width. This indicates that there is no significant wear of the both friction parts, but only minor changes on the surface topography. Because the roughness of the cylinder is higher than that of the disc (see the test methods in additional information section), the applied load is not homogeneously distributed on the apparent contact surface but is mainly carried by the roughest asperities. This fact is clearly visible thanks to the change in colour from green to pink showing the real contact area. The diameter of the X-ray beam for PES analysis was fixed at approximately 50–60 microns to achieve a good signal/noise ratio and a high-energy resolution (Fig. 3).

Before analysis, the cylinder was ultrasonically cleaned with n-heptane. Afterwards, it was left for two weeks in ultrahigh vacuum. We preferred the ultrahigh vacuum solution to heating the sample at 100 C, to desorb contaminants and weakly bonded molecules. We performed high-resolution photoemission surface analysis with two different X-ray energies to vary the depth analysis: a photon energy of 350 eV was used to perform an accurate analysis of the content of carbon-related species at a high depth resolution (an efficient attenuation length (EAL) of approximately 0.65 nm in this case), and the analysis of the samples areas was completed by using a photon flux of 700 eV energy, which allowed us to investigate the distribution of the carbon and oxygen associated species with deeper penetration depth in the coatings (an EAL of approximately 1.4 nm for C1s). Additionally, the variation of the sp^2^/sp^3^ characteristic across the wear scar formed on the ta-C coating was investigated by continuously recording the C1s core level, performed by a line scan throughout the scar with extreme surface sensitivity (using an incident photon energy of 350 eV, as shown in Fig. 3b). The spectroscopic results clearly show the effect of friction on the structure of carbon in the presence of oleic acid.

**Figure 4:**
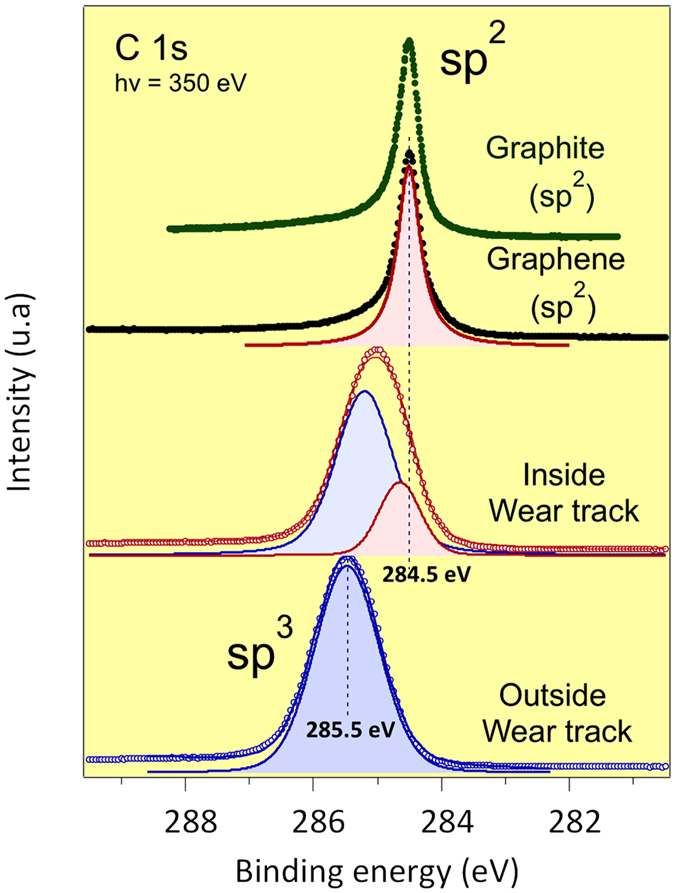
C1s PES spectra recorded inside and outside the ta-C wear scar, using an incident hv = 350 eV. At the top of the figure, the spectra of graphite and graphene samples is also shown as a reference for pure sp2 carbon. All spectra has been taken in the same set-up used for the present study.

**Figure 5:**
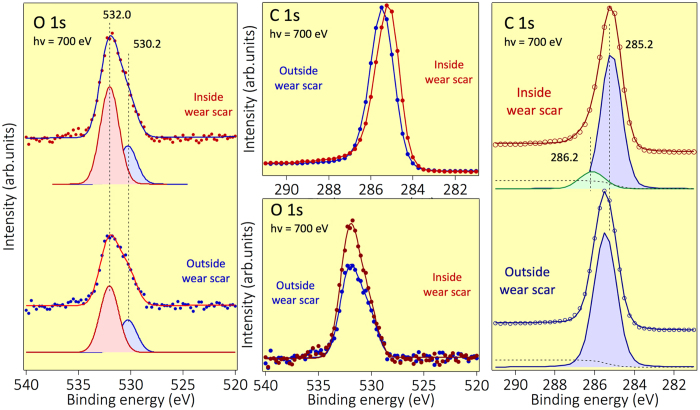
C1s and O1s PES spectra recorded inside and outside the ta-C wear scar, using an incident photon beam of hv = 700 eV. Note that the beamline energy resolution is lower for an incident photon energy of 700 eV than an incident photon energy of 350 eV, therefore, the FWHM of the blue and green components of the C1s peaks are 1.45 eV ± 0.3 eV and 1.8 eV ± 0.3 eV, respectively, which are much larger than those recorded at 350 eV (see text). The FWHM of the blue and pink components of the O1s core level peaks (the left panel of the figure) are both 2.0 eV ± 0.3 eV.

Figure 4 shows selected results of the C1s spectrum; we compare the spectra recorded inside and outside the wear scar, together with the C 1 s recorded on a graphite single crystal and a thin layer of graphene deposited on SiC. First, we observed the presence of a weak peak corresponding to oxidized species^27^,^28^ on the C1s spectrum at 350 eV, typically C-O at 286.2 eV with a FWHM of 0.82 ± 0.1 eV. These oxidized species are also present in small concentration on the spectrum recorded at 700 eV, and the C/O ratio is approximately 10 atomic % in the two cases (Fig. 5).

In the following, we focus on the part of the C1s spectrum corresponding to the C-C bonds in Fig. 4. The detailed examination of the C1s spectrum outside the wear scar is dominated by a strong peak at 285.5 ± 0.2 eV with a FWHM of 1.09 ± 0.2 eV that is attributed to the sp^3^ carbon present on the ta-C surface (including C-C and/or C-H). Inside the wear scar, the C1s peak is clearly shifted by 0.5 eV towards a lower binding energy, and it is composed of two contributions. The result of the optimal fitting confirms that the first contribution inside the scar is at 284.6 eV with a FWHM of 0.7 ± 0.1 eV, and the second one is at 285.2 ± 0.2 eV with a FWHM of 0.8 ± 0.1 eV. The contribution at 284.6 eV can be assigned to the presence of pure sp^2^ carbon such as in graphene, non-planar carbon sheets or graphite. This is in agreement with the C1s peak position of the purely sp^2^ graphene film measured under the same conditions at 284.5 eV (with a FWHM of 0.43 ± 0.1 eV) and with most of the values that can be found in the literature^29^.

Figure 5 shows the same C1s spectra recorded using higher photon energy of 700 eV. Note that the energy resolution of the beamline is lower at the incident photon energy of 700 eV than at 350 eV. The C1s peak is at 285.5 eV outside the wear scar and at 285.2 eV inside the wear scar. The FWHM of the blue and green contributions of the C1s peaks are 1.45 eV ± 0.3 eV and 1.8 eV ± 0.3 eV, respectively, which are much larger than those recorded at 350 eV as previously indicated. In this case, a graphene contribution at 284.5 eV is not required to fit the experimental signal. This is clearly because the analysis depth at 700 eV is much larger (almost twice) than that at 350 eV, and consequently, there is a more important contribution by the carbon from the subsurface of the ta-C coating.

As the typical EAL of X-rays from an incident photon of 700 eV is ~1.4 nm, we can conclude that the graphene-like character of carbon atoms on the surface can hardly be confounded with the presence of bulk graphite crystal (which is a 3D particular arrangement of several graphene sheets). Consequently, the surface termination of the ta-C inside the scar can be mostly associated with a 2D graphene oxide-like film than a 3D graphite-like film owing to the extreme surface sensitivity of the HRPES conducted at low photon energy. We note that it is difficult to differentiate between true graphene-like sheets with only 6-membered rings and non-planar carbon sheets with 5, 6 and 7-membered rings. Hence, the thickness of the graphene-like surface film can be estimated to be at most 1 nm ± 0.5 nm (*i.e*. a maximum of two or three graphene layers), which can hardly be due to 3D stacking in HOPG graphite. On the other hand, there is an indication that the ta-C structure is modified by friction in its subsurface, and this region is at least 2 nm thick. Indeed, the C1s peak energy is at 285.5 eV for an sp^2^ content of about 30% in pristine ta-C and at 284.5 eV for an sp^2^ content of 100% (case of graphene). Assuming a linear relationship between the sp^2^/sp^3^ content and the binding energy of the C1s peak, we can estimate that the subsurface change in the ta-C (with a C1s peak at 285.2 eV) corresponds to an increase of the sp^2^ content of approximately 55% (compared with 30% in the pristine ta-C). Therefore, a thin richer sp^2^-carbon a-C structure has been formed on the top part of the ta-C material under shearing.

We also observe a significant contribution of C-O bonds inside the wear scar from the C1s peak. This is in agreement with previous studies by standard XPS analysis^26^. Figure 5 also shows the O1s core levels recorded inside and outside the scar with higher bulk sensitivity (an EAL of 2 nm). The intensity of the oxygen peak inside with respect to its intensity outside the wear scar increases considerably. In both cases the O1s core level spectrum shows two components at 532.0 ± 0.3 eV and 530.2 ± 0.3 eV, respectively. From the fitting of the peaks, it is evident that only the intensity of the higher binding energy component (the pink peak at the left panel of Fig. 5) increases by almost 50% with respect to the intensity outside the scar. These two components can be assigned as the C-O and C = O species, respectively^27^,^28^. The enrichment in hydroxyl groups on the carbon surface is in good agreement with data previously observed using XPS and ToF-SIMS analysis^26^.

**Figure 6:**
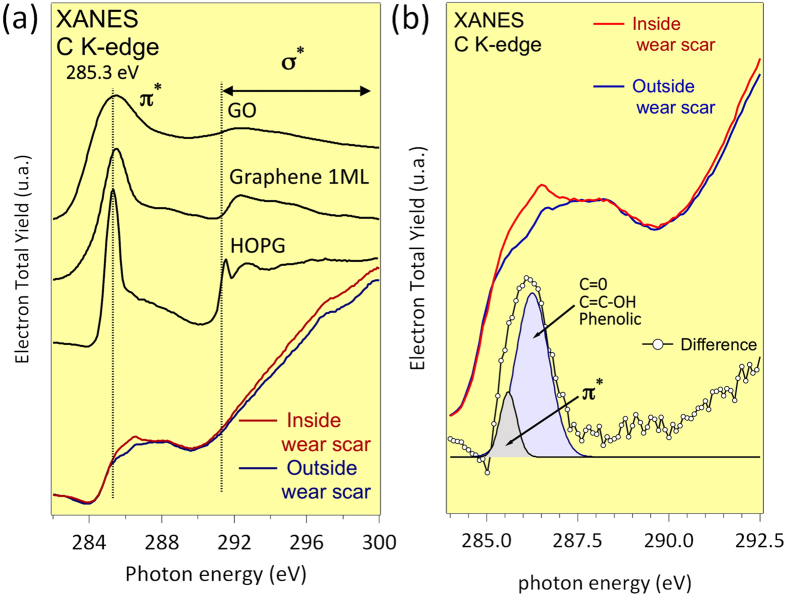
(**a**) XAS spectra of the C K-edge inside and outside the wear scar. At the top of the figure, the spectra of graphite and graphene and graphene oxide samples are plotted (**b**) The spectrum showing the difference is fitted to show the different components.

As it is usual for NEXAFS analysis of carbon-derived films, Highly Oriented Pyrolytic Graphite (HOPG) graphene and GO (Graphene Oxide) are used as the reference material for the calculation and quantification of the sp^2^ and sp^2^/sp^3^ content. This is due to the well-defined electronic structure and almost 100% sp^2^ content of HOPG^30^,^31^. In the case of HOPG, the π* orbitals are aligned normal to the surface, whereas the σ* orbital is localized along the surface. Because the light from the synchrotron source is linearly polarized, the intensities of the π* and σ* transitions are sensitive to the orientation of these orbitals with respect to the polarization vector. At normal incident angles (≈85° with respect to the normal to the surface, considered as 90°), the propagating electric field vector is nearly parallel to the HOPG surface and has a small projection onto the π* orbitals, thus resulting in a weak coupling of the light polarization vector with the π* resonance. Conversely, at glancing angle geometries (≈9°), the electric field vector has a large projection onto the π* orbitals, resulting in the maximum intensity of the π* resonance. To eliminate the orientation effects on the intensity peak associated to the π* state, a XAS study shown in Fig. 6 was performed at an incident X-ray angle of approximately 45° (the magic angle) with respect to the surface normal. In this geometry, the effects of polarization of the synchrotron radiation i.e., the orientation of the graphitic sheets of HOPG, are negligible^30^,^31^,^32^. The total electron yield (TEY) signals were normalized using the intensity of the incident beam obtained from the photoemission yield of a clean Au grid, which is recorded simultaneously while recording the spectra from samples. The normalization was done to eliminate the effects of fluctuations in the incident beam intensity and the absorption features arising from the monochromator.

Figure 6 shows the NEXAFS spectra of the C K-edge recorded outside and inside the wear scar. It also shows the difference obtained by subtracting the two spectra. At higher photon energies above 290 eV, the XAS spectrum is dominated by the 1s-σ * transition of sp^3^ carbon. We will focus on the transitions observed between 285 eV and 290 eV. In this energy range, we observe the weak absorption peak induced by the sp^2^-C 1s-π* transition at 285.4 eV. It is probable that the graphitic material lies at the top surface oriented in the sliding direction, thus the transition of 1s-π* is not fully quenched and the intensity is clearly decreased compared to that of a parallel incidence. Another explanation may be the presence of non-planar sp^2^ sheets (with 5, 6 and 7-membered rings) instead of planar graphene. Other transitions between 286 eV and 288 eV are assigned to 1s-σ* (C-O) corresponding to the epoxy and hydroxyl groups and 1s-σ* (C = O) corresponding to the carbonyl groups. These oxidized species are chemically attached to the basal plane.

To show the consequence of the friction, the spectrum recorded outside the wear scar was subtracted from the spectrum recorded inside the scar (Fig. 6b). The difference clearly shows that two main contributions are enhanced inside the wear scar: the 1s-π* transitions corresponding to sp^2^ carbon near 285 eV and the contribution at approximately 286.5 eV, which typically represents the energies of the 1s-σ* transitions that correspond to oxygen-derived species such as alcohol and phenolic species, according to literature. Although it is difficult to make conclusions from the XAS analysis only, our spectra are in good agreement with those of graphene oxide (GO) published by Da Zhan^32^ and recorded in similar conditions.

**Figure 7:**
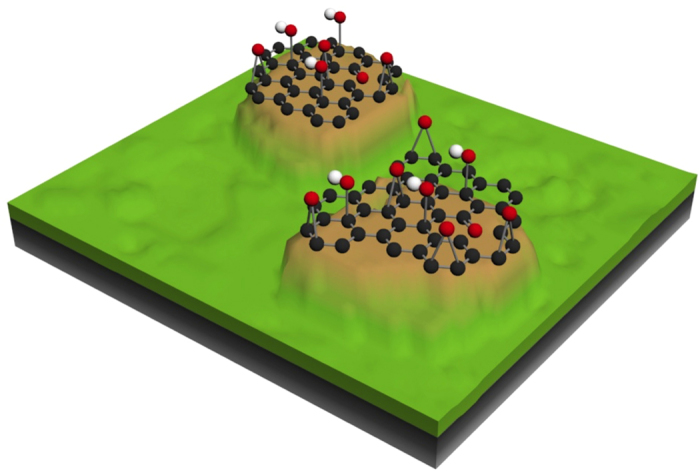
Schematic picture of the ta-C surface after the friction test in the presence of oleic acid (according to PES and XANES analyses). The pink areas correspond to the real contact area between the two antagonists. The green parts are non-contacting areas.

Hence, by combining HRPES and XAS analyses, we have strong indications that the rubbed ta-C surface under lubrication with oleic acid becomes an amorphous sp^2^ rich carbon (a-C) structure terminated with a nanometre-thick film of graphene with a planar structure that is weakly oxidized mainly by OH groups (approximately 10 atomic %). A structure such as this is represented schematically in the picture displayed in Fig. 7. The advantage of this coating compared to the traditional a-C:H one seems to be that tribochemical-reactions are induced by the oleic acid lubricant. Consequently, atomically smooth, partially oxidized graphene-like structures created at the coating top surface afford stable superlow friction regime.

## Discussion

In this work, by using unsaturated oleic acid as a lubricant for ta-C coatings, we show that lubricating graphene/graphene oxide (G/GO) structures can be created *in situ* in the contact zones leading to extremely low friction (Fig. 7). We suspect that the double bond present in the backbone of oleic acid may play a significant role in the formation mechanism of the G/GO structure. This represents a huge advantage for real applications because remarkable performances can be obtained without further addition of lubricating G/GO nanoparticles in the oil. Indeed, in the last few years, different studies have shown that both graphene G and graphene oxide GO nanoparticles dispersed in a suitable lubricant or even water are able to reduce very efficiently the friction and wear of steel-based surfaces. For example, some studies on GO sheets dispersed in a water-based lubricant show that the friction coefficient of a WC/steel contact can be reduced to a value of 0.05, suggesting that the GO sheets act as a lubricant and protective coating^33^,^34^. Moreover, reduced graphene oxide (rGO) coatings have been shown to have superior friction reduction capabilities in water-based lubricants for steel-on-steel applications^35^. The same behaviour of rGO nanoparticles was demonstrated for oil^36^. The same lubricating properties of GO films have been observed for silicon-based MEMS devices^37^. Other nanoparticles like crumpled graphene balls blended to the oil appears also very effective for friction and wear reduction^38^. More generally, graphene-based engine oil nanofluids have been studied for tribological applications in the literature and the friction reduction performance is very promising^39^. So much so that recently, a mixture of nanodiamond particles and graphene nanosheets was shown to form a nanoscroll responsible for the observed superlubricity^40^. AFM experiments^41^, as well as computer simulations^42^,^43^, have also been performed on graphene sheets to explain the microscopic origin of the friction reduction capability. The results show that the graphite mechanical behaviour occurs when several perfect graphene layers are superimposed.

As shown in Fig. 2, there is almost no induction period and friction is achieved immediately in the superlow regime after a few cycles. In a previous paper, we have justified this result by the H/OH-termination of the ta-C surface and the existence of a nanometre-thick oleic acid film between the OH-terminated surfaces in thin film EHL regime^29^. The existence of oleic acid molecules as a thin film further decreases the shear forces because of the low piezo-viscosity coefficient of oleic acid. Moreover the OH-terminated surface being more hydrophilic this facilitates slip at the wall in thin film EHL conditions.

**Figure 8:**
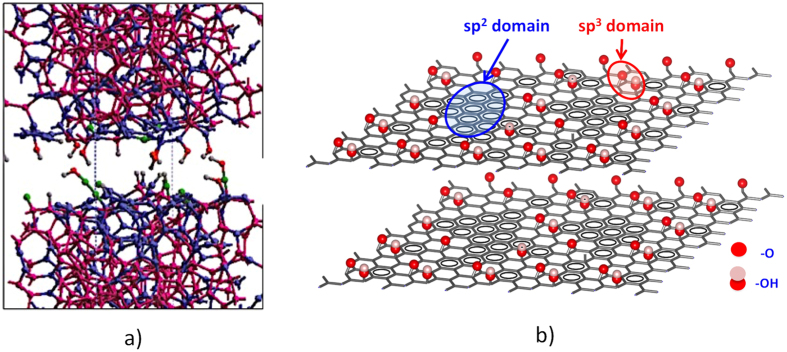
(**a**) Calculated structure of ta-C slabs by ReaxFF reactive force field after saturation of the ta-C/ta-C interface with OH-containing molecules at 27 °C (from ref. 45); (**b**) Schematic representation of graphene oxide layers according to PES and XANES analyses.

In the light of our new results obtained under mixed/boundary regime, the mechanism of superlow friction related to H/OH-terminated DLC can be deeper discussed. In earlier works, we already observed the formation of a-C material enriched in sp2-carbon on the rubbed ta-C surface by EF-TEM and the preferential hydroxylation of carbon atoms by XPS and ToF-SIMS^44^,^45^. We assumed that this was a possible explanation for the low friction when using OH-containing lubricants as glycerol. Studies by Molecular Dynamics combined with ReaxFF reactive force field approach have been done to calculate the DLC structures and the chemical reactions occurring on the surface in the presence of OH-containing molecule. They confirmed the formation of OH-termination on carbon surfaces (see Fig. 8a) and specified that the main role of OH termination is to increase the distance between the carbon atoms of the ta-C substrates and provide sufficient repulsion forces to avoid the formation of covalent C-C bonds across the sliding interface. Compared to only H surface termination, the presence of OH chemical bonds on the carbon surface slightly increases the interfacial distance due to their larger size^45^. This effect is favourable for reducing wear and seizure related to the formation of interfacial C-C covalent bonds. However, the presence of OH-groups in the interfacial region induces some attractive forces between OH and H groups by formation of hydrogen bonds contrary to the main Coulombic repulsive forces between slightly positively charged hydrogen atoms on fully H-terminated carbon surfaces. Moreover H/OH termination slightly increases the atomic roughness of the sliding interface compared with fully H-termination^46^ due to the presence of hook shape terminations (see interfacial region on Fig. 8a). These two facts counteract the beneficial impact of the interfacial distance growth. The numerical calculations effectively showed an increase in the friction force for H/OH termination compared to smoothness fully H-terminated interface^45^ and friction experiments using gas phase lubrication with H_2_ gas and water vapour have also corroborated this result^46^. So, in the light of our new synchrotron data, the model based on the generation of H/OH-terminated a-C surface can be specified with accurate information on the structure of the top layer to explain the superlow friction in mixed/boundary regime with oleic acid.

When analyzing the ta-C surface with PES in this work, we indeed discovered that the very end of the ta-C surface is not completely “amorphous” as predicted in the simulation because it contains domains with too many sp2 carbon atoms (about 90%). The only possibility of a single layer with 90% of sp2 carbon is a graphene-like structure terminated by some oxygenated functions. Figure 8b shows a schematic representation of graphene oxide layers corresponding to our PES quantitative analysis. As it can be seen, OH and C-O-C functions are found *in plane* while acid and carbonyl groups are usually concentrated on the edge. Because the sheets are certainly much larger in the real case, the amount of acid groups is much less in practical cases. We also found by combining PES and XANES that about 10% of residual carbon atoms are mostly OH-terminated (with possibly some ether/epoxide functions). Therefore, there is practically no place for H and C(O)OH terminal bonds in our particular case. In this new situation, there are elements that further contribute to the repulsive forces in the interface due to the occurrence of the graphene-like structure. Long-range Van der Walls attractive dispersion forces between graphitic domains are decreased due to the larger interfacial distance in GO compared with graphite (typically 0.6 nm). The absence of C-H bonds also decreases the occurrence of the attractive hydrogen bonds. Finally, compared to amorphous a-C, the graphene structure reduces the atomic surface roughness and then the friction.

**Figure 9:**
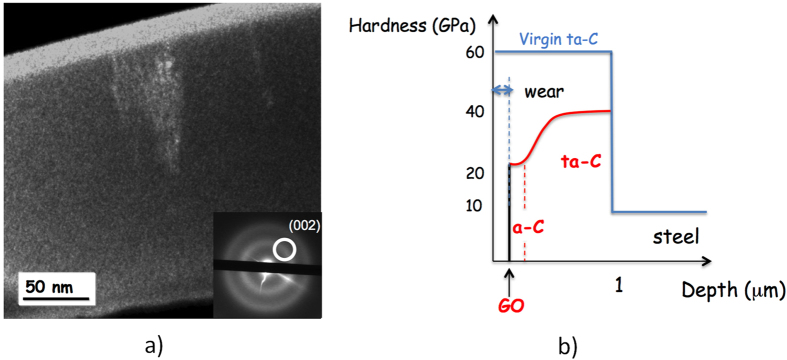
(**a**) TEM Dark Field image of a FIB cross-section of a ta-C coating after lubrication test in glycerol (from ref. 44). The optical diffractogram shows the position of the diaphragm on the (002) ring. A sp^2^-carbon rich film is visible at the top surface; (**b**) schematic view of surface chemistry and hardness changes occurring on the surface of the ta-C coating after the lubrication test.

Summarizing, we propose a slightly different (but not exclusive) model for the formation of the tribofilm:

- Under the effect of high pressure and shear stress during the short running-in period, the ta-C surface becomes smoother and is progressively enriched with sp^2^ carbon, leading to the formation of an amorphous carbon a-C layer. This hybridization change has already been observed in the past with ta-C and nanocrystalline diamond lubricated by glycerol that gives a similar behaviour as oleic acid^44^,^47^. For example, Fig. 9a shows the FIB-EFTEM results for glycerol (a dark field image obtained for the (002) ring of graphite) of the ta-C inside the wear scar, providing clear evidence of the formation of a 30 nm thick sp^2^-rich amorphous layer on the top surface of the ta-C. This layer is not present in the pristine ta-C, which contains only approximately 30% of sp^2^ carbon, and it is formed during the running-in period. The mechanism of the friction-induced change of carbon hybridization from sp^3^ to sp^2^-carbon has also been studied by computer simulations^47^.
- When the sp^2^ content reaches a certain threshold, a graphene-like planar structure spontaneously forms on the a-C surface, and the smoother surface finishing that is obtained decreases the local pressure and may allow oleic acid molecules to reach the contact zone. The role of the double bond present in oleic acid is still unclear but may participate to the aromatization of the top surface.
- Under the effect of oleic acid, the grapheme-like layer is partially oxidized by the tribochemical reaction. Eventually, friction occurs between the graphene oxide nanosheets when the severe boundary/mixed lubrication conditions occurred, leading to a superlow regime (CoF below 0.01). Figure 9b gives a schematic view of the changes that occur in the surface chemistry, hybridization and hardness of the ta-C coating after the lubrication test. Compared to the pristine ta-C coating, the hardness profile that results from the self-adaptation of the coating is optimized for wear reduction, and the presence of GO can decrease the friction efficiently. Comparatively, a softer hydrogenated DLC coating does not show this self-adaptation of the surface properties during friction.

**Figure 10:**
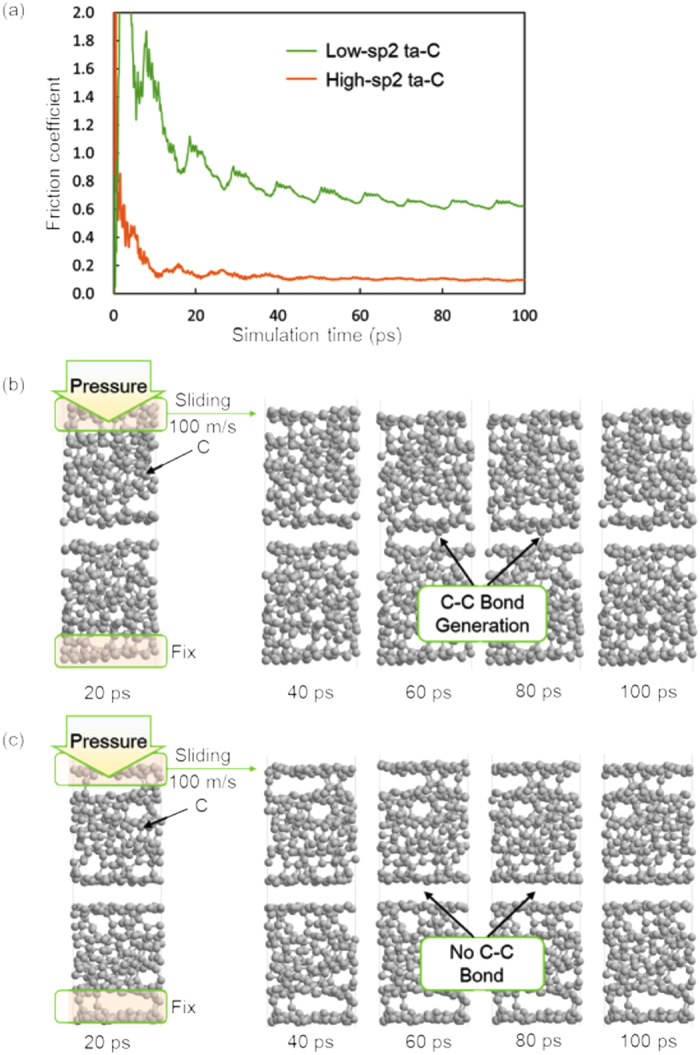
(**a**) Friction coefficients of the low-sp^2^ and high-sp^2^ a-C models under a contact pressure of 0.5 GPa. Snapshots of the friction simulations for the (**b**) low-sp^2^ and (**c**) high-sp^2^ a-C models at 20, 40, 60, 80 and 100 ps.

**Figure 11:**
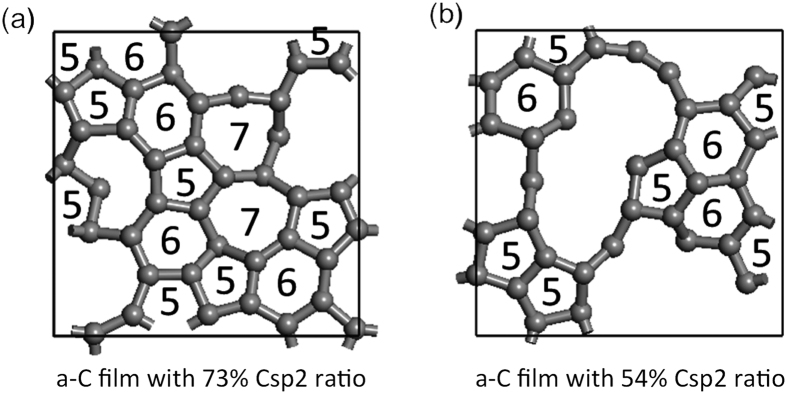
Computed extreme surface top-views of the (**a**) high-sp^2^ and (**b**) low-sp^2^ a-C models at 100 ps. The numbers in the figures show the position of the 5-, 6-, and 7-membered rings.

To determine the possibility of this mechanism and particularly the existence of step 2, we perform friction atomistic simulations with two different amorphous carbon coatings, an a-C model with a sp^2^/sp^3^ ratio of 54% and a second one with a sp^2^/sp^3^ ratio of 73%, by employing our tight-binding quantum chemical molecular dynamics (TB-QCMD) code “Colors” (see computational methods in additional information section). The simulations are performed under a contact pressure of 0.5 GPa at 300 K, and the top layer of the upper slab is forcibly slid over with a horizontal velocity of 100 m/s. Figure 10a compares the friction coefficients for the low-sp^2^ and high-sp^2^ a-C models. The friction coefficient is 0.62 and 0.1 for the low-sp^2^ and high-sp^2^ a-C models, respectively. It clearly indicates that the friction coefficient drastically decreases with an increasing sp^2^/sp^3^ ratio in the carbon structure. Figure 10b shows snapshots of the friction simulations for the low-sp2 a-C model at 20, 40, 60, 80, and 100 ps under a contact pressure of 0.5 GPa. We observe that the high friction coefficient of the low-sp^2^ a-C model occurs because of the generation of the C-C bonds across the sliding interface. Figure 10c shows snapshots of the friction simulations for the high-sp2 a-C model at 20, 40, 60, 80, and 100 ps under a contact pressure of 0.5 GPa. In that case, the sliding appears much smoother and no C-C bond is generated across the sliding interface leading to a low friction coefficient. Figure 11 shows the top-view of the atomic planes in the two cases at 100 ps. It is clear that at the atomic level, carbon rings are present with different numbers of carbon atoms (5, 6 and 7), and the atoms are in almost the same plane with the same sp^2^ hybridization for the high-sp^2^ a-C model. In contrast, for the low-sp^2^ a-C model, although some carbon rings are formed, a number of carbon atoms stay out of the interfacial plane in a sp^3^-hybridization state.

Thus, we theoretically confirm that a high-sp^2^ carbon content in the surface of the carbon coating indicates a lower friction property compared to a low-sp^2^ carbon content owing to the absence of C-C bonds at the friction interface and the formation of a quasi-flat graphene-like interface containing different carbon rings. In a second step we will add OH termination and possibly oleic acid molecules in the interfacial zone. However performing such computer simulations with QCMD approach is much more complicated and time-consuming.

## Conclusion

Steel friction pairs coated with ultra-smooth ta-C coating show superlubricity behaviour under oleic acid lubrication in the mixed regime. The friction coefficient is below 0.01 in the thin-film EHL/mixed regime and it is below 0.03 in the boundary regime. By coupling advanced extreme surface analysis and computer simulations, we show that the mechanism of friction reduction below 0.01 is related to the tribo-formation of a graphene-like structure at the top surface (thickness less than 1 nm). Moreover, the rubbed sub-surface is enriched with sp^2^-hybridized carbon, such as in the a-C material, which certainly eases the tribochemical-formation of the carbon rings present in the graphene-like structure.

Based on this insight, we propose a new mechanism to explain the formation of the tribofilm. First, during the running-in period, the top surface of ta-C is enriched with sp^2^ carbon owing to the relatively high shear stress and becomes slightly smoother. Second, once the sp^2^ carbon content reached a certain threshold (probably around 60% according to the simulation), the 2D structure can be preferentially formed at the top of the colliding asperities. Eventually, the graphene can be slightly oxidized by the oleic acid decomposition. The role of oleic acid in the graphene formation, and especially the presence of the double bond, needs to be further investigated because, unsaturated fatty acids are known to produce aromatized chemical species. Moreover advanced analyses of the a-C:H surface showing much higher friction would be interesting and is currently under consideration.

## Methods

The hydrogen-free ta-C coating was deposited on polished hardened bearing AISI 52100 steel by a filtered arc-ion plating deposition process (PVD). It presents a very smooth surface without droplets leading to a roughness comparable to that of usual hydrogenated a-C:H coating produced by plasma-enhanced chemical vapour deposition process (PECVD). The ta-C coating displays very high hardness and Young’s modulus of 60 GPa and 650 GPa, respectively measured by nanoindentation (see details in Supplementary Information section).

The unidirectional rotating cylinder-on-disc sliding experiements were conducted in the following manner. The fixed cylinders, measuring 9 mm in diameter and 9 mm in length, were made of hardened bearing steel (AISI52100) and were polished in order to obtain a surface roughness below RMS of 15 nm (root mean square roughness). The rotating 3 mm-thick disc has a diameter of either 33 mm or 18 mm depending on the lubrication regime. It was also made of same AISI 52100 steel and polished to a surface roughness below RMS 2.6 nm in order to get a very low surface roughness after the deposition of DLC coating. Contact at the sliding interface was in the shape of line under moderate Hertzian pressure of about 70 MPa. Lubrication was provided by a few droplets (0.01 ml) of the lubricant on the disc at room temperature. The sliding speed was varied in a range of 0–0.1 m/s for the tribological experiments.

The XAS and HRPES measurements were carried out at station ANTARES of the SOLEIL synchrotron radiation source facility, France.

The absorption data was acquired in the total electron yield (TEY) mode by collecting electron emission from the sample and were normalized to the signal from a gold covered grid recorded simultaneously. The resolution of the beam line is 50 meV at the CK-edge. Data analysis was performed after background correction and subsequent decomposition into several Gaussian peaks.

The HRPES analysis was performed at the same experimental station suing 350 eV and 700 eV incident photon energies. The chemical compositions, thickness and chemical bonding states inside and outside the scar were systematically investigated. In order to avoid beam damage to the samples, no ion beam sputtering was performed on the investigated samples. The photoemission spectra were obtained at a Room Temperature of the sample using a hemispherical analyser Scienta R4000. The high-resolution core level spectra were acquired at 20 eV pass energy and 50 meV energy increments. For the correction of binding energy of samples, the C1s core level peak was calibrated to the gold 4 f peak occurring at 84 eV. The high-resolution spectra were fitted using multiple Voigt functions, corresponding to the various moieties present in the samples.

In this paper, we employ our TB-QCMD simulator to explore the bond formation and bond breaking dynamics induced by electron transfer during tribological phenomena. This method was already employed to analyze the superlow friction mechanism on the electronic- and atomic-scale^48^ and the software package “Colors” is applied to the tribochemical reaction dynamics of the DLC films. All the details about Hamiltonian function are described in the Supplementary Information section.

## Additional Information

**How to cite this article:** Bouchet, M. I. D. B. *et al*. Diamond-like carbon coating under oleic acid lubrication: Evidence for graphene oxide formation in superlow friction. *Sci. Rep.* **7**, 46394; doi: 10.1038/srep46394 (2017).

**Publisher's note:** Springer Nature remains neutral with regard to jurisdictional claims in published maps and institutional affiliations.

## Supplementary Material

Supplementary Information
