# Supplementary material for: Diamond-like carbon coating under oleic acid lubrication: Evidence for graphene oxide formation in superlow friction
Source: Sci Rep. 2017 Apr 12;7:46394. doi: 10.1038/srep46394 (PMC5388889; doi:10.1038/srep46394)
Supplement: Supplementary Information [file srep46394-s1.pdf]

Diamond-like carbon coating under oleic acid lubrication: Evidence for graphene oxide formation in superlow friction.

Maria Isabel De Barros Bouchet[1], Jean Michel Martin[1], José Avila[2], Makoto Kano[3], Kentaro Yoshida[3], Takeshi Tsuruda[4], Shandan Bai[5], Yuji Higuchi[4], Nobuki Ozawa[4], Momoji Kubo[4] and Maria C. Asensio[2]*

[1)] University of Lyon, Ecole Centrale de Lyon, LTDS, 69380, Ecully, France

[2)] Antarès Beamline, Synchrotron SOLEIL, University Paris-Saclay, France

[3)] Kanagawa Industrial Technology Center 705-1 Shimo-Imaizumi, Ebina, Kanagawa, Japan

[4)] Institute for Materials Research, Tohoku University, 2-1-1 Katahira, Aoba-ku, Sendai 980-8577, Japan

[5)] New Industry Creation Hatchery Center, Tohoku University, 6-6-10 Aoba, Aramaki, Aoba-ku, Sendai 980-8579, Japan

SUPPLEMENTARY INFORMATION

Test method

The unidirectional rotating cylinder-on-disc sliding tests were conducted in the following way. The cylinders, measuring 9 mm in diameter and 9 mm in length, were made of hardened bearing steel (AISI52100) and were polished in order to obtain a surface roughness below Rq of 15 nm (root mean square roughness). The 3 mm thick disc has a diameter of either 33 mm or 18mm depending on the lubrication regime. It was also made of same steel and polished to a surface roughness below Rq 2.6 nm. The ta-C coating was produced by a Filtered PVD process and presents a very smooth surface without droplets leading to a roughness comparable to that of a-C:H (produced by PECVD process). The ta-C coating has very high hardness and Young's modulus of 60 GPa and 650 GPa, respectively measured by nanoindentation. Table S1 gives the main properties of the DLC used.

Table S1 Properties of DLC coatings

	Steel (uncoated)	ta-C	a-C:H
Coating method		(PVD Filtered)	PACVD
Coating thickness (μm)		0.3	1.1
Disc roughness Rq (nm)	2.6	3.7	3.5
Cylinder Rq (nm)	12	16.1	13
Composite roughness (nm)	9	10	8
Hardness (GPa)	10	61	20
Young modulus (GPa)	200	650	250

The cylinder was fixed in order to prevent it from rotating and was pressed against the rotating disc, as shown in Figure S1. Contact between the cylinder and the disc is linear under Hertzian pressure of around 70 MPa and the width of the rectangular wear scar is calculated to be 50 microns. Before starting to slide, a few droplets (0.01ml) of the lubricant were provided by gently wetting the disc. Temperature of the whole system was fixed at ambient temperature (25 °C). The relative humidity plays an important role in this experiment because of the small quantity of lubricant in the contact area. RH will be specified in each test.

First, a "decreasing speed test" was conducted. The speed was progressively reduced step by step from 100 mm/s to 0.01mm/s, with holding the speed for 5 minutes at each step. The results are expected to make clear the different friction property between a-C:H and ta-C according to the sliding speed.Second, using a new friction pair, the "constant sliding speed test" was conducted to focus on the tribofilm formation in most interesting cases. The sliding test was conducted at 50 mm/s for 900 s under oleic acid lubrication to investigate the difference in tribofilm chemistry on the sliding tracks. XPS analyses were performed either on the cylinder or disc wear scar.

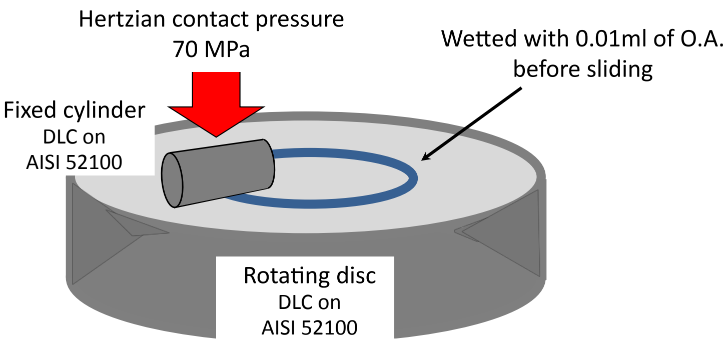

Fig. S1: Cylinder-on-disc sliding test[26]

The lubricant is pure oleic acid ($C_{18}H_{34}O_2$) that contains a double bond in the backbone and the carboxylic group –COOH at the extremity. The viscosity of oleic acid at ambient temperature (25 C) is 33.0 mPa.s and the viscosity-pressure coefficient is about 7 GPa^{-1}.

Computational Methods

The friction simulations were performed with our TB-QCMD code "Colors", where the Hamiltonian has the following forms

$$H_{rs} = \begin{cases} -I_r, & \text{if } r = s, \\ \frac{1}{2} K_{rs} S_{rs} (H_{rr} + H_{ss}), & \text{otherwise,} \end{cases} \quad (1)$$

$$K_{rs} = \{1 + k_{rs}(1 - \Delta^4) + \Delta^2\} \exp[-\delta_{rs}\{r_{rs} - (d_r + d_s)\}] \quad (2)$$

$$\Delta = \frac{H_{rr} - H_{ss}}{H_{rr} + H_{ss}} \quad (3)$$

The diagonal matrix element, H_{rr}, is defined as the negative of valence electron ionization potential, I_r; that is, $H_{rr} = -I_r$. The off-diagonal term H_{rs} is calculated from equation (1), where S_{rs} is the overlap integral matrix. In equation (2), r_{rs} is the distance between two atoms to which the molecular orbitals belong, k_{rs} and δ_{rs} are the positive parameters for the tight-binding approximation, and d_r is the radius of each orbital. The total energy, E_{total}, of the systems is given by the kinetic energy, the eigenvalue, ε_k, the repulsive potential, E_{rep}, and Lennard-Jones (LJ) potential, E_{LJ}.

$$E_{total} = \sum_{i=1}^{N} \frac{1}{2} m_i v_i^2 + \sum_{k}^{occ} \varepsilon_k + \sum_{i=1}^{N} \sum_{i<j}^{N} E_{rep}(r_{ij}) + \sum_{i=1}^{N} \sum_{i<j}^{N} E_{LJ}(r_{ij}) \quad (4)$$

$$E_{rep}(r_{ij}) = b_{ij} \exp\left(\frac{a_{ij} - r_{ij}}{b_{ij}} \right) \quad (5)$$

$$E_{LJ}(r_{ij}) = \begin{cases} U_0, & 0 \le r_{ij} < r_a \\ C_1 \cdot cos\left(\frac{\pi(r_{ij}-C_2)}{2C_3}\right) + C_4, & r_a \le r_{ij} < r_0 \\ 4\epsilon\left[\left(\frac{\sigma}{r}\right)^{12} - \left(\frac{\sigma}{r}\right)^{6}\right], & r_0 \le r_{ij} < r_b \end{cases} \tag{6}$$

Here, k is the index for the molecular orbital, and i and j are indices for the atoms in the system. The inter-atomic parameters a_{ij} and b_{ij} are related to the size and stiffness of the atoms i and j. The parameters for the calculation are shown in Table S2 of supplementary information. In equation (6), U_0 takes a constant value of 1.16 kcal/mol. $\varepsilon = 0.414$ kcal/mol and $\sigma = 3.36$ Å are LJ parameters for C atoms. C_1, C_2, and C_3 are constants for height, position, and periodicity of trigonometric function potential. Here, $C_1 = 0.787$ kcal/mol, $C_2 = 3.02$ Å, and $C_3 = 0.38$ Å. C_4 is defined by half of sum of U_0 and $-\varepsilon$, and $C_4 = 0.373$ kcal/mol. r_a, r_0 and r_b are defined by the LJ parameter of σ. $r_a = 0.9\ \sigma$, $r_0 = 2^{1/6}\ \sigma$, and $r_b = 2.5\ \sigma$. r_b is the cut-off radius for the calculation.

To construct the ta-C models, first we employ the diamond bulk model with vacuum layer, which contains 216 C atoms in total. This diamond model is heated to a temperature of 4500 K for 10 ps, then is cooled to 300 K. According to the above procedures, we create two friction simulation models of low-sp2 (54%) and high-sp2 (73%) ta-C. The friction coefficient μ, is given by, $\mu = F_x$-averaged/F_z-averaged, where F_x and F_z represent the sums of the horizontal and perpendicular forces with respect to the top layer of upper slab, respectively. F_x-averaged and F_z-averaged are the averaged values of F_x and F_z for every step, respectively.

Table S2 Parameters for TB-QCMD calculation

Atom	I^a [eV]		ζ^b [Å^{-1}]	
	s orbital	p orbital	s orbital	p orbital
C	21.40	12.80	1.975	2.100

[a]Ionization energy from the valence orbital. [b]Exponent Slater-type atomic orbital.

Atom pair	a_{ij} [Å]	b_{ij} [Å]
C-C	2.245	0.160

Orbital pair	κ_{rs}	δ_{rs} [Å^{-1}]
C(s)-C(s)	1.200	0.130
C(s)-C(p)	0.750	0.130
C(p)-C(p)	1.200	0.130

Soft x-ray absorption and high-resolution photoemission spectrocopies

The XAS and HRPES measurements were carried out at station ANTARES of the SOLEIL synchrotron radiation source facility, France, under ring operating conditions of 2,5 GeV electron energy, an injection currents of 500 mA and "Top-up" mode. The radiation was monochromatized using a plane-grating monochromator (PGM), which is characterized by a slitless entrance and the use of two Varied Linear Spacing (VLS) gratings with a Variable Groove Depth (VGD) along the grating lines. The absorption data was acquired in the total electron yield (TEY) mode by collecting electron emission from the sample and were normalized to the signal from a gold covered grid recorded simultaneously. The resolution of the beam line is 50 meV at the CK-edge. Data analysis was performed after background correction and subsequent decomposition into several Gaussian peaks. The HRPES analysis was performed at the same experimental station suing 350 eV and 700 eV incident photon energies. The chemical compositions,

thickness and chemical bonding states inside and outside the scar were systematically investigated. . In order to avoid beam damage to the samples, no ion beam sputtering was performed on the investigated samples.

The high-resolution core level spectra were acquired at 20 eV pass energy and 50 meV energy increments. For the correction of binding energy of samples, the C1s core level peak was calibrated to the gold 4f peak occurring at 84 eV. Peak fitting of the core level spectra was carried out using vendor provided software, Igor, after a Shirley background subtraction. The high-resolution spectra were fitted using multiple Voigt functions, corresponding to the various moieties present in the samples. In figure 4 during the fitting procedure, an iterative cycle was carried out. First, the position and Full Width at Half Maximum (FWHM) of each peak was fixed, while the peak areas were freely iterated. Second, the positions and FWHM were varied in a least-square refinement.
